# Genetics and Adaptation of Soybean Cyst Nematode to Broad Spectrum Soybean Resistance

**DOI:** 10.1534/g3.116.035964

**Published:** 2017-01-03

**Authors:** Michael Gardner, Robert Heinz, Jianying Wang, Melissa G. Mitchum

**Affiliations:** Division of Plant Sciences and Bond Life Sciences Center, University of Missouri, Columbia, Missouri 65211

**Keywords:** *Heterodera glycines*, nematode, plant resistance, soybean, virulence

## Abstract

The soybean cyst nematode (SCN) *Heterodera glycines* is a major threat to soybean production, made more challenging by the current limitations of natural resistance for managing this pathogen. The use of resistant host cultivars is effective, but, over time, results in the generation of virulent nematode populations able to robustly parasitize the resistant host. In order to understand how virulence develops in SCN, we utilized a single backcross BC_1_F_2_ strategy to mate a highly virulent inbred population (TN20), capable of reproducing on all current sources of resistance, with an avirulent one (PA3), unable to reproduce on any of the resistant soybean lines. The offspring were then investigated to determine how virulence is inherited on the main sources of SCN resistance, derived from soybean lines Peking, PI 88788, PI 90763, and the broad spectrum resistance source PI 437654. Significantly, our results suggest virulence on PI 437654 is a multigenic recessive trait that allows the nematode to reproduce on all current sources of resistance. In addition, we examined how virulence on different sources of resistance interact by placing virulent SCN populations under secondary selection, and identified a strong counter-selection between virulence on PI 88788- and PI 90763-derived resistances, while no such counter-selection existed between virulence on Peking and PI 88788 resistance sources. Our results suggest that the genes responsible for virulence on PI 88788 and PI 90763 may be different alleles at a common locus. If so, rotation of cultivars with resistance from these two sources may be an effective management protocol.

The soybean cyst nematode (SCN) *Heterodera glycines* remains a major threat to soybean production, and continues to spread wherever soybeans are grown worldwide (Tylka and Marett 2014). Yield losses attributed to SCN on an annual basis are estimated at >$1.2 billion (Koenning and Wrather 2010). To date, the most effective way of managing this pathogen has been through the use of host resistance. However, widespread deployment of resistant cultivars has led to the selection of virulent SCN adapted to the resistance utilized (Niblack *et al.* 2006). To address this, rotations of different resistant cultivars are currently recommended. However, much remains unknown about the mechanism of nematode adaptation to resistant cultivars, including the virulence genes responsible for overcoming resistance. Precise details about the heritability and independence of nematode virulence genes are needed to inform rotation schemes of resistant cultivars that minimize selection for SCN populations adapted to multiple resistant varieties.

The first study on the inheritance of SCN genes responsible for virulence noted that the ability of the nematode to reproduce on the resistant soybean cultivars PI 88788 and Pickett was inherited independently (Triantaphyllou 1975, 1987). Following this discovery, the *ror* (for reproduction on a resistant host) genes were identified using a controlled SCN crossing strategy (Dong and Opperman 1997). These *ror* genes allowed some SCN populations to reproduce on PI 88788 (*ror-1*) and PI 90763 (*ror-2*). *ror-1* and *ror-2* were shown to be inherited in a Mendelian fashion, independently of each other, in a dominant and recessive manner, respectively. At the time of their discovery, the resistance genes in soybean remained unknown, so the role these nematode *ror* genes have in the interplay between nematode and host was not characterized.

It was also noted that SCN populations adapted to reproduce on one resistant soybean line were able to reproduce better on some, but not all, other resistant lines. For example, it was shown that culturing nematodes on soybean lines PI 88788, PI 209332, or PI 548316 (Cloud) resulted in SCN populations that grow well on these lines, but not on the soybean lines PI 548402 (Peking), PI 90763, and PI 89772 (Anand and Brar 1983; McCann *et al.* 1982; Young 1994). Based on these observations, two genetic groups of soybeans were proposed, with group one containing PI 88788, PI 209332, and Cloud, while group two contains Peking, PI 90763, and PI 89772 (Luedders and Dropkin 1983; Colgrove and Niblack 2008). In recent years, much has been discovered about the genes responsible for resistance in both groups, specifically underlying two quantitative trait loci named *Rhg1* (for resistance to *H. glycines*), and *Rhg4* (reviewed in Mitchum 2016). Resistant soybean lines in group one only require *Rhg1* for resistance to SCN HG type 0 (Race 3) populations. These soybean lines have a variable number of tandem repeats containing a set of four dissimilar genes (*i.e.*, 7–10 copies) at *Rhg1* (Cook *et al.* 2012; Lee *et al.* 2015). Three of the genes within the repeat were shown to be required for resistance. By contrast, soybean lines in group two contain only a few repeats (*i.e.*, 1–3 copies) at *Rhg1* (Cook *et al.* 2012; Lee *et al.* 2015), but also require a second QTL, *Rhg4*, where a single serine hydroxymethyltransferase (SHMT) gene controls resistance to SCN HG type 0 (Race 3) populations (Liu *et al.* 2012). While great strides have been made to identify some of the soybean genes controlling resistance, the mechanisms by which virulent SCN overcome these two major types of resistance, often referred to as Peking-type and PI 88788-type, remain unknown.

PI 437654 is the first reported soybean line to show resistance to nematodes adapted to each of the two soybean resistance groups (Anand *et al.* 1985). This broad resistance is a stark contrast to previous known resistance, and may provide a valuable alternative to rotating between the other two resistance groups. To date, very few field populations of SCN have been identified that are able to successfully reproduce on PI 437654, although the numbers have been increasing with the increased use of this source of resistance. However, synthetic inbred SCN populations, such as LY1 and TN20, that can reproduce on PI 437654 have been developed. LY1 was generated from a mass mating of SCN Race 2 (HG Type 1.2-) females with SCN Race 5 (HG Type 2-) males (Arelli *et al.* 2009). The TN20 inbred SCN population was generated by single cyst-descent of LY1 on PI 437654 in 1999, and has since been mass-selected on PI 437654 (Atibalentja *et al.* 2005). Interestingly, SCN populations that can reproduce on PI 437654 also show increased reproductive success on all other resistant indicator lines used in the HG type test (HG Type 1–7; Niblack *et al.* 2002), and so continuous usage of this cultivar is not recommended (Niblack 2005). Because of the increased use of PI 437654-derived resistance in commercial soybean lines, and identification of these virulent individuals in SCN field populations (Niblack *et al.* 2006), it is important to understand how this resistance is related to PI 88788 and Peking, the two main sources of resistance currently in use, and, subsequently, how SCN virulence on PI 437654 is related to virulence on these sources.

In this study, we set forth to describe the genetic factors that allow the SCN population TN20 to parasitize PI 437654 using a controlled crossing method. Once this was understood, we put it into context with SCN reproduction on other resistant soybean lines using secondary selection. Together, this information provides novel insights from the nematode perspective on overcoming host resistance, and the first possible evidence for multigenic virulence in SCN.

## Materials and Methods

### Parental lines

**Figure 1:**
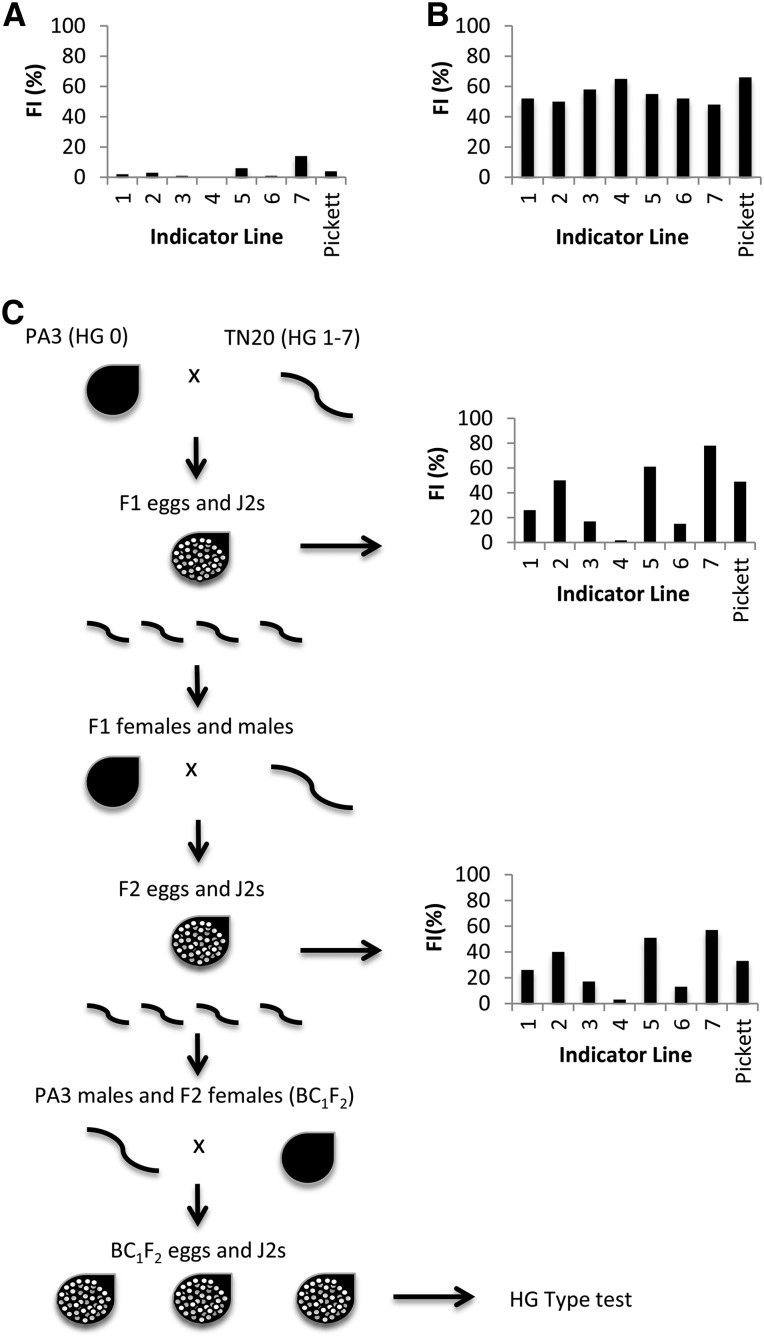
Crossing strategy for determining inheritance of virulence genes. (A) Avirulent females (PA3) were crossed with (B) highly virulent males (TN20), and the F_1_ offspring were phenotyped using an HG type test. (C) F_1_ males and females were then crossed, and the resulting F_2_ generation was backcrossed to PA3 in a BC_1_F_2_ strategy [drawing adapted from Dong and Opperman (1997)]. The F_2_ population was also outselected on eight resistant soybean indicator lines to generate inbred lines MM7–MM14.

Inbred soybean cyst nematode (*H. glycines* Ichinohe) lines PA3 and TN20 were used in these experiments. PA3 and TN20 were maintained on the SCN-susceptible cultivar Williams 82 (W82) and PI 437654, respectively. The female index (FI), where FI = (mean number of females on a test soybean line)/(mean number of females on the standard susceptible) × 100, is used to describe the percentage of an SCN population that can reproduce on a resistant line. The *ror* genes are present in extremely low frequency in the PA3 population, which results in a female index (FI) of <10 on any one of the seven plant introductions (PI) used in the HG type test (Niblack *et al.* 2002). These include PI 548402 or Peking (1), PI 88788 (2), PI 90763 (3), PI 437654 (4), PI 209332 (5), PI 89772 (6), and PI 548316 or Cloud (7). The *ror* genes are present in high frequency in the TN20 population, which results in a FI of >50 on all seven indicator lines. The host ranges of these populations were tested in the greenhouse, with four replications, and 1000 eggs per plant, on SCN-susceptible cultivar Lee74, the seven HG test indicator lines, and Pickett (formerly used in the race designation scheme). PA3 is a HG Type 0 (Race 3) population. TN20 is a HG Type 1–7 (Race 4) population (Figure 1).

### Crosses and F_1_ generation testing

Reciprocal cross-matings between PA3 and TN20 were conducted using a bulk sample of females from one line, and a bulk sample of males from another line in soil. Juveniles were hatched from eggs of freshly harvested females of each inbred line, and inoculated onto 7-d-old W82 seedlings in 400-ml tri-cornered plastic beakers. After 7–10 d, the roots were carefully washed free of soil, and the plants were suspended in half-strength Hoagland’s solution, and continuously aerated by bubbling air from aquarium pumps at 27°. Males began to emerge from the roots 14 d after inoculation, and sunk to the bottom of the tube. They were collected on a 400-mesh sieve every day between 15 and 20 d postinoculation. Plants with virgin females were transferred to pots, and inoculated with males; 15 d after mating, the females were harvested, bulked and crushed to release F_1_ eggs. Eggs of the F_1_ generation from reciprocal crosses were advanced to the F_2_ generation on Lee74 and used for host range tests on Lee74, Peking, PI 88788, PI 90763, PI 437654, PI 209332, PI 89772, Cloud, and Pickett. Tests were conducted according to Brown *et al.* (2010), with four replications and 1000 eggs per plant. F_1_ generation females from each host were counted.

### Mating and backcrossing

We used a backcross 1 F_2_ (BC_1_F_2_)-derived mating strategy (Dong and Opperman 1997). The female and recurrent parent PA3 was nonparasitic (avirulent) on resistant hosts, and the male parent, TN20, was parasitic (virulent). In the BC_1_F_2_ strategy, the individual eggs, juveniles and virgin females segregate for parasitism:nonparasitism at the F_2_ generation. The backcross was made at this stage, and individual fertilized females were selected, disrupted to release eggs, and individual batches of eggs were inoculated to the susceptible soybean Lee74 to generate 90 progeny lines. After several generations of amplification, individual plants were harvested, and cysts were isolated. These individual bulk progeny lines were used for host range testing.

### Cross-population selections

Eggs of the F_2_ generation from reciprocal crosses were used for host range tests as described above. F_2_ generation females from each host were counted, crushed to release eggs, and inoculated back to Peking, PI 88788, PI 90763, PI 437654, PI 209332, PI 89772, Cloud, and Pickett. These outselected populations, named MM7–MM14 were inbred for at least 72 generations, following which host-range tests were performed according to above.

### Secondary selection

Eggs from the outselected populations MM7, MM8, and MM10 were used to inoculate the susceptible soybean variety W82, and a different resistant soybean line. MM7 was inoculated to PI 88788, MM8 to Peking, and MM10 to both PI 88788 and Peking. The resulting offspring were propagated under the same conditions on the same host for a total of 12 generations. At zero, six, and 12 generations, host range tests were conducted on Lee, Peking, PI 88788, PI 90763, and PI 437654.

### Data availability

Strains are available upon request. Supplemental Material, Table S1 contains modified host range test results for segregating progeny lines. 

## Results

### Crosses, F_1_, and F_2_ generation host range tests

Reciprocal crosses were conducted between the PA3 population, which does not reproduce on resistant cultivars, and the TN20 population, which can reproduce on all sources of resistance. The PA3 population had a FI of <10% on the resistant indicator lines, whereas TN20 had a FI of nearly 50% or more on all resistant indicator lines at the start of this study. Figure 1 depicts the F_1_ generation host range test results from reciprocal crosses of PA3 × TN20. The F_1_ generation from PA3 × TN20, and its reciprocal cross-produced a FI of >10% on Peking, PI 88788, PI 90763, PI 209332, PI 89772, PI 548316 (Cloud), and Pickett, indicating that at least one virulence gene for reproduction on each of these resistant hosts in TN20 is dominant or partially dominant. The relatively high FI (49–78%) of the F_1_ generation on PI 88788, PI 209332, Cloud and Pickett, consistent with the TN20 parent population, suggests that virulence is dominant. The intermediate FI (15–26%) of the F_1_ generation on Peking, PI 90763, and PI 89772, suggests that virulence is not controlled by a single dominant or recessive gene. In contrast, no reproduction was observed in the F_1_ generation on PI 437654. Therefore, virulence in TN20 to PI 437654 is recessive. Results of reciprocal crosses indicated that there is no preferential effect of the donor parent (*i.e.*, sex linkage) on inheritance of virulence genes consistent with prior studies (Dong and Opperman 1997). Host range test results of the F_2_ generation were similar, except there was a slight increase in the FI from 0 to 3% on PI 437654.

### Progeny lines and parasitism gene number

Ninety individual bulk progeny lines were established from [(TN20 × PA3) × PA3]. Results from the parasitism tests are depicted in Table 1 and Table S1. BC_1_F_2_-derived single female descent lines should segregate into a 3:1 ratio for parasitism:nonparasitism for a single gene trait, or 15:1 for two separate genes. The progeny lines yielded 68 parasitic and 22 nonparasitic phenotypes on PI 88788. The observed ratio was not significantly different from the 3:1 ratio (*P* = 0.903) (Table 1). These results show that there is a single dominant gene in TN20 that confers the ability to parasitize PI 88788. This gene may correspond to *ror-1* described by Dong and Opperman (1997). Evaluation of the same progeny lines for the ability to parasitize PI 90763 and Peking yielded similar results. In both cases, the observed ratio was not significantly different from the 3:1 ratio (*P* = 0.114 for PI 90763; *P* = 0.068 for Peking). Prior studies reported *ror-2* and *ror-3*, recessive genes for virulence on PI 90763 and Peking, respectively (Dong and Opperman 1997; Dong *et al.* 2005). Results from the progeny lines also revealed an unexpected phenomenon. Only seven of the 90 progeny lines showed any reproduction on PI 437654, and the highest FI observed was only 2%. These data indicate that virulence on PI 437654 is unlikely to be controlled by an independent recessive gene, but rather a combination of the existing *ror* genes in the nematode.

**Table 1:** Host range test of PA3 × TN20 segregating progeny lines on Peking, PI 88788, PI 90763, and PI 437654

Cross*^a^*	PI*^b^*	Lines*^c^*	P:Np*^d^*	Exp-rt*^e^*	χ^2^	*P*
[(PA3 × TN20) × PA3]	Peking	90	30:60	3:1	3.333	0.068
[(PA3 × TN20) × PA3]	PI 88788	90	68:22	3:1	0.015	0.903
[(PA3 × TN20) × PA3]	PI 90763	90	16:74	3:1	2.504	0.114
[(PA3 × TN20) × PA3]	PI 437654	90	0:90	3:1	270	<0.001

a TN20 was the parasitic and PA3 was the nonparasitic *H. glycines* inbred line on Peking, PI 88788, PI 90763, and PI 437654. The BC_1_F_2_ mating strategy used PA3 as the female and recurrent parent in a single backcross.

b Plant introduction.

c Number of progeny lines from the mating.

d Numbers of parasitic (P) and nonparasitic (Np) lines on the indicator lines. Parasitic lines were scored based on a >10% female index.

e Expected segregation ratio for a single gene.

### Host ranges of outselected inbred populations

**Figure 2:**
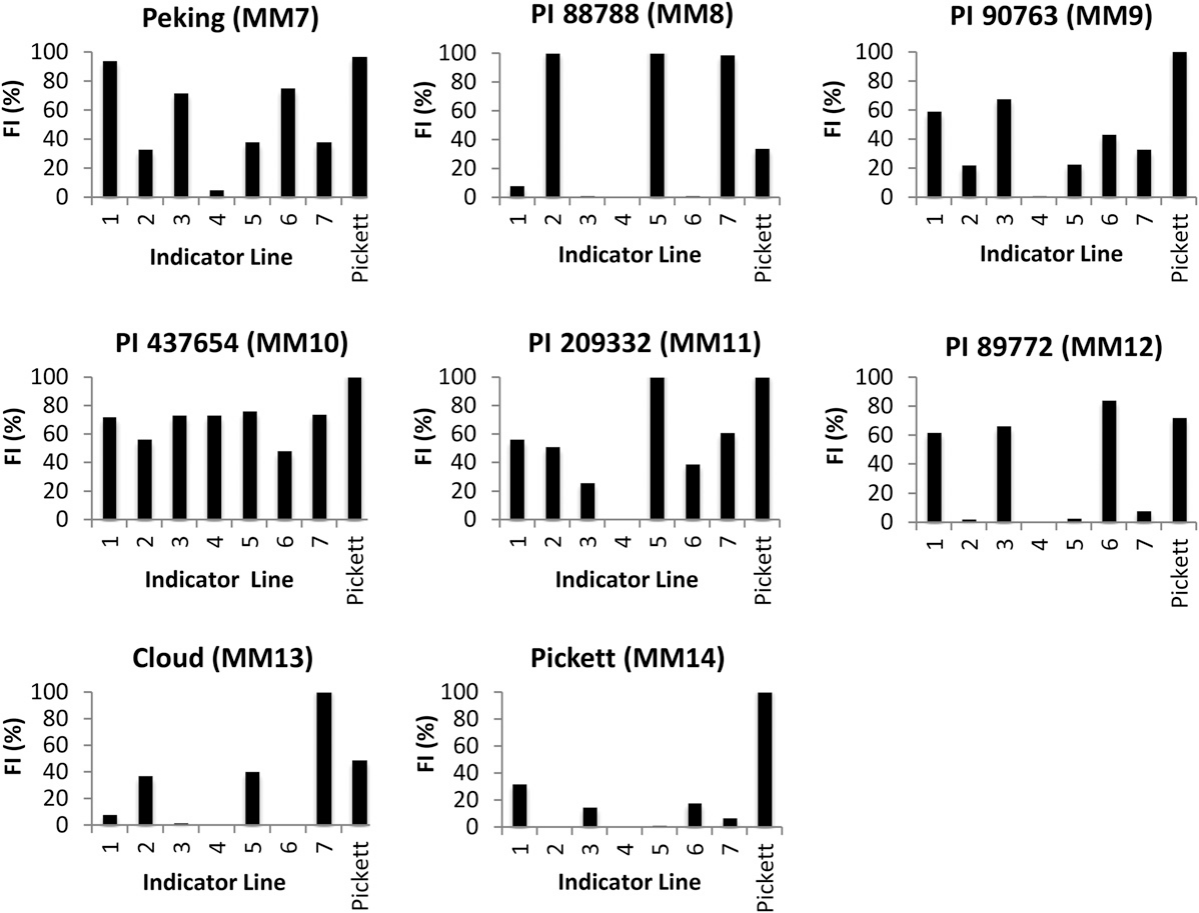
Outselected inbred populations from a cross between PA3 × TN20. Females successful on each of the lines in the F_2_ SCN HG type test were used to generate SCN inbred populations MM7–MM14 through mass selection for a minimum of 72 generations on each indicator line.

Figure 2 depicts the host range test results of inbred lines MM7–MM14 developed from the PA3 × TN20 cross-population outselected at least 72 generations on each of the seven HG indicator lines and Pickett. Following selection, the populations fell primarily into two groups. The first group had elevated virulence (>40% FI) on Peking, PI 90763, and PI 89772. The second group had elevated virulence on PI 88788, PI 209332, and Cloud. Cross-populations MM7 (Peking), MM9 (PI 90763), and MM12 (PI 89772) fall into the first group while MM8 (PI 88788), MM11 (PI 209332), and MM13 (Cloud) fall into the second group. The exceptions to this were the population selected on PI 437654 (MM10), which maintained high virulence on all indicator lines screened, and the population selected on Pickett, which maintained a low level of virulence on Peking, PI 90763, and PI 89772 (Figure 2).

### Secondary selection

**Figure 3:**
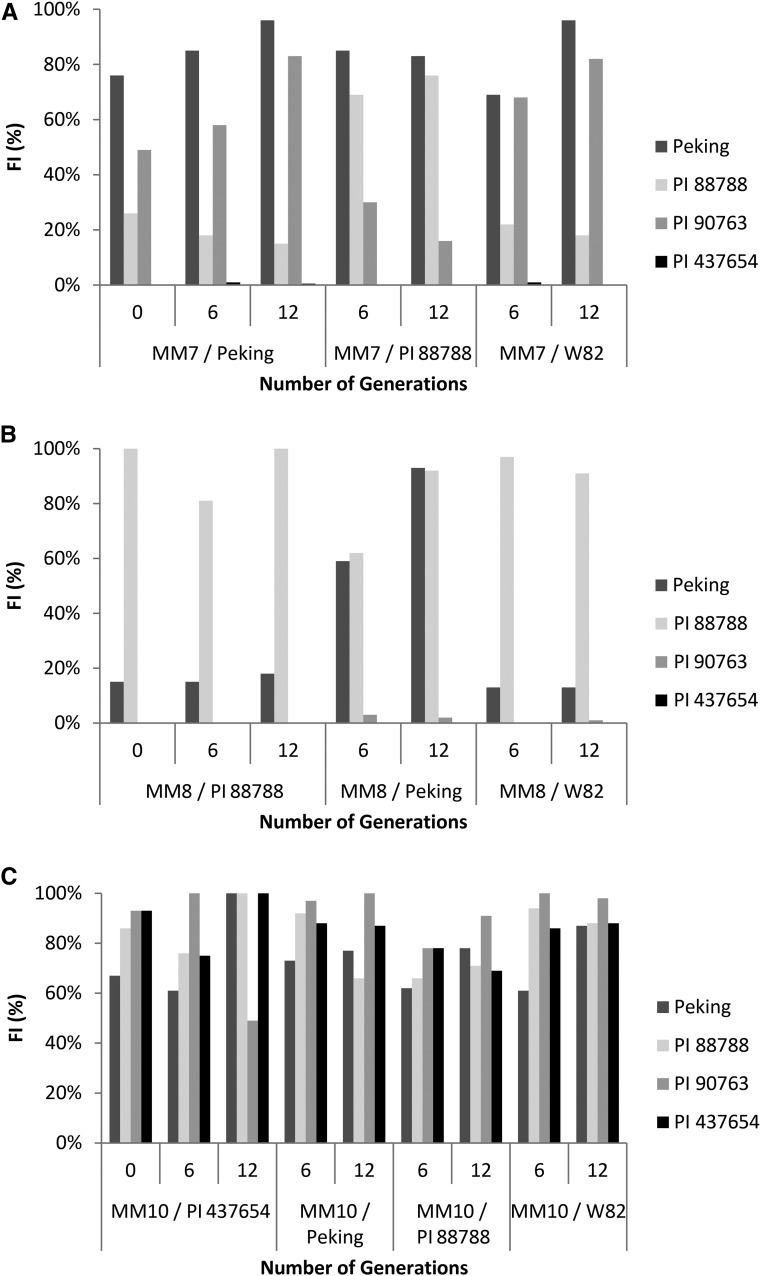
Secondary selection of outselected inbred populations. Outselected inbred populations MM7 (A), MM8 (B), and MM10 (C) were placed under secondary selection on different soybean resistance lines or on susceptible soybean. HG type tests were conducted at zero, six, and 12 generations to assess shifts in virulence as a result of secondary selection.

We then used the MM7, MM8, and MM10 inbred SCN populations in a secondary selection experiment. MM7, which was originally selected on Peking, was placed under secondary selection on PI 88788 and the SCN susceptible cultivar W82; MM8, originally selected on PI 88788, was placed under secondary selection on Peking and W82; MM10, originally selected on PI 437654 was placed under secondary selection on Peking, PI 88788, and W82. At intervals of six and 12 generations, the virulence of these populations was assessed using a modified HG type test restricted to four of the normal seven indicator lines. The MM7 population selected on PI 88788 gradually increased in virulence, while still maintaining high virulence on Peking. In addition, MM7 virulence on PI 90763 was significantly decreased after 12 generations of selection on PI 88788 (Figure 3A). Importantly, no change in virulence was observed in MM7 selected on the susceptible soybean cv. W82. The MM8 population selected on Peking maintained high virulence on PI 88788 and greatly increased in virulence on Peking, but only showed a minor increase in virulence on PI 90763 (Figure 3B). Like MM7, no change in virulence was observed in MM8 selected on susceptible soybean W82. Finally, MM10, which was originally selected on PI 437654 maintained high virulence on all indicator lines after 12 generations of secondary selection on PI 88788, Peking, or W82 (Figure 3C).

## Discussion

Previous reports have implicated independent genes inherited in both a dominant and a recessive manner in SCN virulence on a variety of resistance sources (Dong and Opperman 1997). The offspring of the PA3 × TN20 F_1_ generation support these previous findings, with high FI on PI 88788, where the *ror-1* gene is inherited in a dominant manner, and recessive genes, *ror-2* and *ror-3*, for reproduction on PI 90763 and Peking, respectively (Dong *et al.* 2005). However, nothing was known about the inheritance of virulence genes on PI 437654-derived resistance, or the impact of sex linkage upon virulence gene inheritance. Our tests sought to describe the SCN genes responsible for virulence on PI 437654, and try to distinguish if these genes are independent of those required for virulence on PI 88788 and Peking resistance. To determine if there was any relationship between sex of the parent in the initial cross, and the virulence profile of the offspring, we performed the cross with the virulent parent as both a male and a female. The resulting F_1_ and F_2_ offspring had a nearly identical HG type, indicating that the virulence genes were likely inherited independently of sex.

Following the initial cross between TN20 and PA3, almost no females were observed within the F_1_ generation on PI 437654. Within the F_2_ generation, there were slightly more females on PI 437654, but still many fewer than on other resistant soybean lines. This indicated that the virulence gene(s) responsible for reproduction on PI 437654 is inherited in a recessive manner. By limiting the population to those individuals successfully able to infect PI 437654, we were able to generate a robust HG type 1–7 population. The fact that these individuals were able to reproduce on PI 88788 and Peking, despite never having been exposed to these sources of resistance, was an indication that the gene (s) responsible for virulence on PI 437654 also conferred virulence on these other sources. This is also consistent with the mass-mating studies of Race 2 and Race 5 SCN populations to generate the LY1 inbred population (Arelli *et al.* 2009). Race 2 can reproduce on Peking and PI 88788, but not PI 90763, whereas Race 5 can reproduce on PI 88788, but not Peking and PI 90763. Following selection of the cross-population on Hartwig, which derives resistance from PI 437654, a robust HG Type 1–7 population was generated. The striking absence of virulent individuals on PI 437654 in host range tests of TN20 × PA3 segregating progeny lines suggests that virulence on PI 437654 may be multigenic, and therefore specified by a combination of *ror* genes inherited in a recessive fashion, allowing it to reproduce on all current sources of resistance.

The inbred SCN populations outselected from the F_2_ population on each of the indicator lines (MM7–MM14) normally used in a HG type test allowed us to follow nematode adaptation to each resistance source. In the outselected SCN populations, we observed distinct 1.3.6 or 2.5.7 HG types for those populations selected on PI 88788, PI 89772, Cloud, and Pickett following closely with patterns observed in previous research (Dong *et al.* 2005; Colgrove and Niblack 2008). By contrast, populations outselected on Peking, PI 90763, and PI 209332 showed a 1.2.3.5.6.7 HG type with elevated FI on indicator lines 1, 3, and 6 for Peking and PI 90763 outselected populations, and elevated FI on indicator lines 5 and 7 for the PI 209332 outselected population. SCN HG type 1.2.3.5.6.7 populations contain a combination of the genes required for virulence on Peking-type and PI 88788-type resistances yet still lack virulence on PI 437654. Also of note are the results observed for the outselected population on Pickett. Pickett was used in the original race determination scheme for SCN populations, but was dropped with the adoption of the HG type system because it derives resistance from Peking, and was thus deemed redundant (Niblack *et al.* 2002). Based on the results of this study, Pickett has likely inherited genes for resistance to HG Type 0 (Race 3), but not other resistance genes in the Peking background (Rao-Arelli *et al.* 1992). This likely explains why field populations are commonly identified that can reproduce on both Peking and Pickett (Races 2, 4, 9, and 14), Pickett and not Peking (Races 5, 6, 10, and 15), but not the opposite combination (Peking and not Pickett; Races 11, 12, 13, and 16).

Secondary selection of the three virulent inbred SCN populations MM7, MM8, and MM10 also revealed interesting connections between mechanisms of virulence on the PI 88788, Peking, and PI 90763 resistance sources. Following secondary selection on PI 88788, MM7 shifted from its initial strong virulence on Peking and PI 90763 to be highly virulent on Peking and PI 88788. Meanwhile, MM7 lost virulence on PI 90763, indicating reciprocal secondary selection on this source of resistance. This was again visible in the MM8 population as secondary selection on Peking increased virulence on Peking, but not PI 90763. Both of these observations support the conclusion that there is an antagonistic relationship between virulence on PI 88788 and PI 90763. Our results support earlier work of Luedders and Dropkin (1983), who reported the same phenomena for different SCN populations on Cloud and PI 89772, or PI 88788 and PI 90763. The latter authors suggested that some SCN genes for reproduction on genetically different soybeans may be alleles, although linkage is possible. Further evidence exists when examining selection of a Race 5 SCN isolate on Bedford (PI 88788-derived resistance) and Cordell (Peking and PI 90763-derived resistances) soybean lines (Young 1994). SCN selected on Cordell for 14 generations lost virulence on PI 88788-derived resistant soybean, while gaining virulence on Peking- and PI 90763-derived resistant soybean. Here, the authors again suggest that the genes responsible for virulence on PI 88788 and PI 90763 may be different alleles at a common locus. If so, rotation of cultivars with resistance from these two sources may be an effective management protocol. The secondary selection studies also show a lack of reciprocal secondary selection between the PI 88788 and Peking resistance sources. Following secondary selection, both MM7 and MM8 maintained high FI on PI 88788 and Peking, but not PI 90763. This is congruent with observations of secondary selection on PI 89772 using SCN populations initially selected on PI 209332, where secondary selection did not completely eliminate virulence on the initial soybean host, while gaining virulence on the secondary host (Luedders 1985).

In summary, this study illustrates how nematodes that have adapted to PI 437654 are able to overcome all sources of resistance, and highlights the potential threat of this type of population to soybean production. PI 437654 was the first source of resistance identified to have resistance to Races 3, 4, and 5. Anand *et al.* (1985) proposed that the use of this line in cultivar development could eliminate the need to combine resistance genes from PI 88788 and PI 90763 to achieve multi-race resistance (Anand *et al.* 1985), in contrast to generating cultivars with resistance from PI 88788 and PI 90763, and using these in rotation. Our results suggest that HG type 1–7 SCN populations are likely to develop through a combination of *ror* genes, and, therefore, generating cultivars with resistance from PI 88788 and PI 90763, and using these in rotation may be a better strategy. In addition, all virulent (MM7, MM8, and MM10) populations retained their high FI on resistant soybean, even following secondary selection on the susceptible soybean W82 for 12 generations, indicating that virulence genes remain fixed in a population in the absence of selection pressure.

Together with previous studies, the research presented here has implications for the management of SCN using natural resistance. Currently, extension agencies generally advise to rotate between resistant soybean lines with different sources of resistance when possible, to slow the build-up of virulent SCN populations (Niblack and Tylka 2012). This may be advisable in some situations and not others, as effective management is likely to depend on the resistant cultivar-SCN population combination. As the molecular details of the soybean-SCN interaction are elucidated through the cloning of soybean resistance genes (*Rhg*) and SCN virulence genes (*ror*) genes, management recommendations can be tailored for more strategic management of field populations.

## Supplementary Material

Supplemental material is available online at www.g3journal.org/lookup/suppl/doi:10.1534/g3.116.035964/-/DC1.
