# Supplementary material for: Genetics and Adaptation of Soybean Cyst Nematode to Broad Spectrum Soybean Resistance
Source: G3 (Bethesda). 2017 Jan 3;7(3):835–41. doi: 10.1534/g3.116.035964 (PMC5345713; doi:10.1534/g3.116.035964)
Supplement: Supplementary file 1 [file 835TableS1.docx]

Table S1. Modified host range test results for 90 segregating progeny lines. (.xlsx, 27 KB)

<http://www.g3journal.org/lookup/suppl/doi:10.1534/g3.116.035964/-/DC1/TableS1.xlsx>
